# Role of *Salvinia molesta* in biodecolorization of methyl orange dye from water

**DOI:** 10.1038/s41598-020-70740-5

**Published:** 2020-08-19

**Authors:** Israa Abdulwahab Al-Baldawi, Siti Rozaimah Sheikh Abdullah, Asia Fadhile Almansoory, Nur ’Izzati Ismail, Hassimi Abu Hasan, Nurina Anuar

**Affiliations:** 1 Department of Chemical and Process Engineering, Faculty of Engineering and Built Environment, Universiti Kebangsaan Malaysia, 43600 UKM Bangi, Selangor Malaysia; 2 Department of Biochemical Engineering, Al-Khwarizmi College of Engineering, University of Baghdad, Baghdad, Iraq; 3 Department of Ecology, Science Collage, Basrah University, Basrah, Iraq

**Keywords:** Biotechnology, Environmental sciences, Engineering

## Abstract

In the present study, the potential of *Salvinia molesta* for biodecolorization of methyl orange (MO) dye from water was examined. Six glass vessels were filled with 4 L of water contaminated with MO with three concentrations (5, 15, and 25 mg/L), three with plants and another three without plant as contaminant control. The influence of operational parameters, including initial dye concentration, pH, temperature, and plant growth, on the efficacy of the biodecolorization process by *S. molesta* was determined. Temperature and pH was in the range of 25–26 °C and 6.3 to 7.3, respectively. Phytotransformation was monitored after 10 days through Fourier transform infrared (FTIR) spectroscopy, and a significant variation in the peak positions was demonstrated when compared to the control plant spectrum, indicating the adsorption of MO. The highest biodecolorization was 42% in a 5 mg/L MO dye concentration at pH 7.3 and at 27 °C. According to the FTIR results, a potential method for the biodecolourization of MO dye by *S. molesta* was proven. *Salvinia molesta* can be successfully used for upcoming eco-friendly phytoremediation purposes for dye removal.

## Introduction

The quick growth of the textile industry has led to the accumulation of several organic pollutants in surface bodies of water, which has indirect or direct contrary effects on the environment^1,2^. Increasing industrialization and urbanization has been resulting in the discharge of waste into the environment, which in turn is creating more pollution. The release of contaminated effluents from many textile industries undesirably disturbs water resources, soil potency, aquatic life, and ecosystems. The release of these dye-containing wastes to the ecosystem will cause a decrease in dissolved oxygen concentration, blocked sunlight penetration, and reduced photosynthesis, resulting in toxic environmental conditions^3^. Conventional technologies for the treatment of dye discharges through physicochemical methods alone generally cannot comply with stringent environmental regulations. In addition, there are other drawbacks of these physicochemical treatments, including high energy consumption, the requirement of chemical addition, and the generation of hazardous sludge as a by-product^4,5^. Biomass is a persistent and renewable source on earth. Thus, researchers are attempting to use it in the field of green biotechnology. Biomass, such as charcoal and activated carbon, is a popular adsorbent for water purification^6,7^. In comparison, phytoremediation is a promising approach to protect the aquatic environment, based on a plant’s ability to degrade, absorb, transform, and remove organic pollutants in water^8,9^. This technology has been widely used to degrade or remove different types of pollutants, including heavy metals^10–13^, hydrocarbons^14–17^ and nutrients^18–21^. Phytoremediation of textile dyes has been an alternative treatment due to its inexpensive and eco-friendly approach and the lower amount of sludge produced^22,23^. Few studies report the removal of textile dyes using floating plants.

Phytoremediation is selected based on numerous benefits, such as cost-effectiveness and its aesthetic view^24^. In addition, it does not generate hazardous materials and it can achieve sustainable development goals^25^. The potential of different plants to degrade dyes has been proven. Kagalkar et al.^26^ concluded that *Blumea malcommi* was able to degrade Direct Red 5B dye, and Abdulqader et al.^27^ used the tropical emergent plant *Scirpus grossus*, with 64% decolourization of 200 mg/L MO in synthetic wastewater. Also, Davies et al.^28^ demonstrated the degradation of textile factory discharge containing Azo Acid Orange 7 dye using a vertical flow constructed wetland planted with *Phragmites australis.* Another study by our team^23^ showed the successful transformation of methylene blue using *Azolla pinnata*, with 85% removal. Phytoremediation was employed to removal toxic dyes and to perform decolorization with biological degradation processes^29^.

In the present study, the floating plant *Salvinia molesta* was selected to explore the ability of the plant to decolourize methyl orange (MO) dye. *Salvinia molesta* is usually found in static or quiet waters such as lakes, rivers, wetlands, and canals^30^. The habitat of *Salvinia molesta* is in tropical, sub-tropical or warm temperate areas of the world such as Malaysia. The plant structure of *S. molesta* is a free-floating fern with rootless stems and hairy roots. It can grow up to 20 cm long and the fern colour is light green to medium green colour, with brownish edges when mature^31^. Much research deals with floating plants, which are highly tolerable to many environmental contaminants, such as heavy metals and hydrocarbons^32,33^, but few studies have shown that the plants have the ability to biodecolorize textile dyes^2,34^. In the present study, the aim of this research was to explore the ability of *Salvinia molesta* to decolourize MO dye in synthetic wastewater.

## Materials and methods

### Lab-scale arrangement for decolorization of methyl orange dye

**Figure 1:**
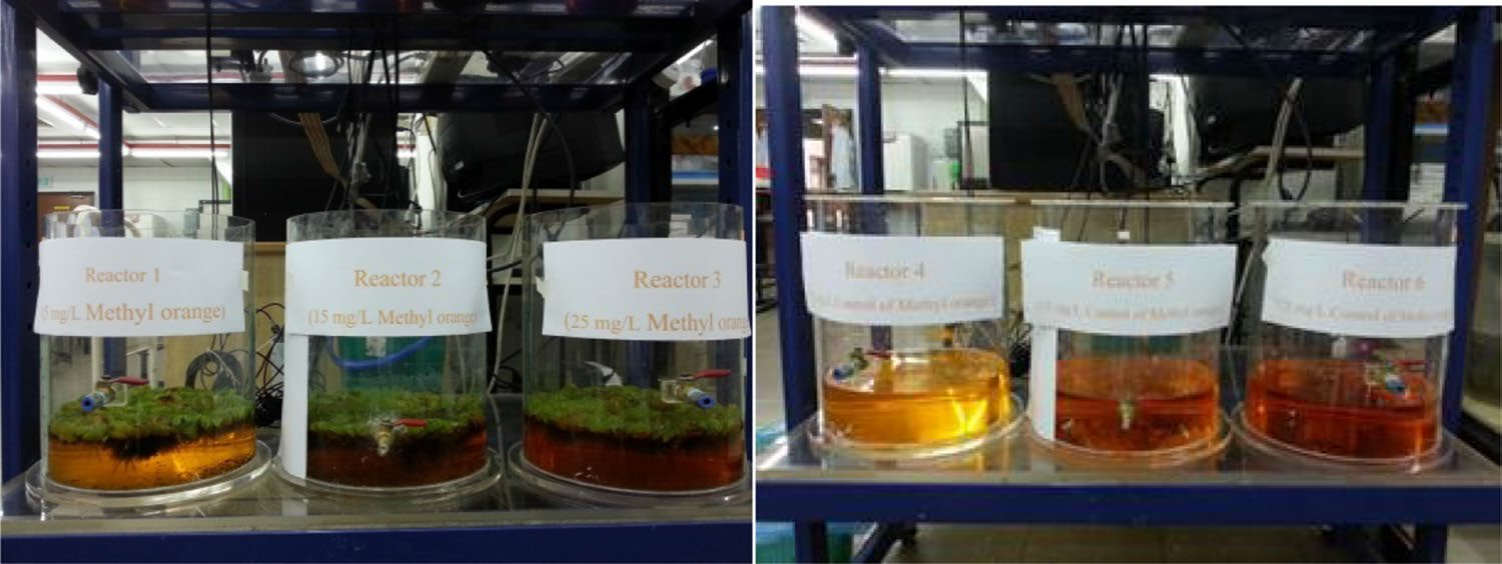
Glass vessel setup for decolorization of methyl orange (MO) (Glass vessel 1 = 5 mg/L MO + *S. molesta*; Glass vessel 2 = 15 mg/L MO + *S. molesta*; Glass vessel 3 = 25 mg/L MO + *S. molesta*; Glass vessel 4 = control glass vessel for 5 mg/L MO; Glass vessel 5 = control glass vessel for 15 mg/L MO; Glass vessel 6 = control glass vessel for 25 mg/L MO).

Decolorization experiments were carried out in glass vessels using the floating plant *S. molesta*. MO dye purchased from R&M Chemicals Marketing of Chemical Reagent Company (U.K.) was utilised as a classic dye in this study. Six glass vessels, with diameters of 25 cm and heights of 30 cm, were used, and each was filled with 4 L of synthetic water contaminated with MO dye with three different concentrations (5, 15, and 25 mg/L). Approximately 150 g of fresh floating plant of *S. molesta* were placed in three glass vessels, as shown in Fig. 1, and the other three glass vessels represent contaminant control. The dye strength in water samples and standards were determined through adsorption values with an Ultraviolet/Visible (UV/Vis) spectrophotometer (Lambda 35 UV/V) at 465 nm with distilled water used as the blank.

After measurement of the dye concentration, the samples were returned back to the glass vessel immediately. The parameter percent decolorization is defined in Eq. (1) as the following:

1 $${\text{Decolourization}}\,\left( \% \right) = \frac{{{\text{MO}}_{0} - {\text{MO}}_{{\text{d}}} }}{{{\text{MO}}_{0} }}$$

with, MO_0_ is the absorbance value of the samples at day 0 and MO_d_ is the absorbance value of the samples on the sampling day.

### Physical parameters of aquatic media

The physical parameters of the wastewater, including temperature (°C), pH, dissolved oxygen (DO, mg/L), and oxidation reduction potential (ORP, mV), were recorded to evaluate conditions for dye removal. The physical parameters were monitored on day 0, 1, 2, 3, 5, 8, and 10 using a multi-probe IQ 150 (I.Q Scientific Instruments, U.K.) to measure the pH, ORP, and temperature measurements. To record the DO, a dissolved oxygen sensor (GLI International, Model 63, U.S.A.) was used.

### Growth observation of *Salvinia molesta*

Plant growth observations were obtained over a 10-day period on days 0, 5, and 10. Two plants were taken from each glass vessel on the sampling day to record plant wet and dry biomass. It offers a clear advantage in understanding the ability of the floating plant to survive and tolerate MO dye in phytoremediation mechanisms. The relative growth rate (RGR) was determined based on the increase in dry biomass weight (BW) after 10 days of investigation, using Eq. (2)^35^ as follows:

2 $$RGR\,\left( {{\text{day}}^{ - 1} } \right) = \frac{{\left[ {\ln W_{f} - \ln W_{i} } \right]}}{{{\text{Day}}}}$$

with, *W*_*i*_ is the initial weight (g) and *W*_*f*_ is the final weight (g) after exposure to the dye contaminant.

### Analysis of phytotransformation by *Salvinia molesta*

Phytotransformation was measured using Fourier transform infrared (FTIR) spectroscopy techniques (NICOLET 6,700 Spectrophotometer, USA) in the mid-IR region of 400–4,000 cm^−1^ with 16 scan speed. At the end of exposure, the whole plant of *S. molesta* was first dried in an oven at 120 °C for 1–3 days until constant mass achieved and blended. One g of dried sample of S. molesta was sent to FTIR analysis. The specimens were blended with spectroscopically pure KBr in the proportion of 5:95. The pellets were located in specimen holder and then analysed^36^. The FTIR spectra between the MO powder, dried plant control, and dried plant after 10 days of dye exposure experiments were compared to confirm the biodegradation of the dye into various metabolites.

### Analysis of variance

Statistical analyses were conducted using Statistical Product and Service Solutions (SPSS) 16.0 for Windows. Two-way analysis of variance (ANOVA) was selected to determine the role of the plant in removal of MO from water. In addition, tests of between-subjects effect were analysed for MO removal and plant growth with treatment (with and without plant), time, and MO concentrations. Duncan’s multiple range tests were applied to assess statistical differences in the relative growth rate factor at the probability level of 0.05, unless otherwise stated. The samplings were triplicated, and the results are presented as means ± standard deviation.

## Results and discussion

### Physical states of treatment

The variations in temperature, pH, ORP, and DO during the exposure times were recorded to show the conditions of the selected floating plant of *S. molesta* to remove MO dye from water. For the three MO concentrations of 5, 15, and 25 mg/L, temperature and pH were almost equal in both treatments (with plants and without plants are shown in Table 1). The experiments were carried out under lab condition with a temperature range of 25–27 ± 0.5 °C. The solution pH in the glass vessel with plants and without plant were in the range of pH 6.3–7.3, demonstrating the suitable pH needed for growth and activity of S*. molesta*, especially for dye removal. According to a study conducted by Yaseen and Scholz^37^, pH has no obvious influence in phytoremediation with *S. molesta*. However, Khataee et al.^2^ determined that a pH value of 6.5 was the optimal decolourization pH. In this experimental the pH and temperature reported values were in natural environment which is satisfactory for the MO decolorization process.

**Table 1:** Physical condition of glass vessels. Mean SD (n = 3).

Parameter	With plants	Without plants
T (°C)	(25–26) ± 0.5	(26–27) ± 0.5
pH	(6.3–7.3) ± 0.5	(6.3–7.3) ± 0.5
DO (mg/L)	(4–5) ± 1	(3–4) ± 1
ORP (mV)	(7 to − 50) ± 10	(50 to − 50) ± 10

For DO measurement, it can be observed that the effect of *S. molesta* on the physical conditions varied between 4 and 5 mg/L for the three MO concentrations (5, 15, and 25 mg/L). The initial ORP of the glass vessels with plants was 7 ± 10 mV, which then decreased to (− 50) ± 10 mV, showing a significant influence of the plant, which plays an important role in the performance of decolourization, with a decrease in dissolved oxygen and lower ORP values due to the activity of the bacteria. In contrast, for the glass vessel without plant the DO and ORP were even lower, ranging between (3–4) ± 0.5 mg/L and (50 to − 50) ± 10 mV due to the MO dye and the absence of the plant.

### Plant growth

**Figure 2:**
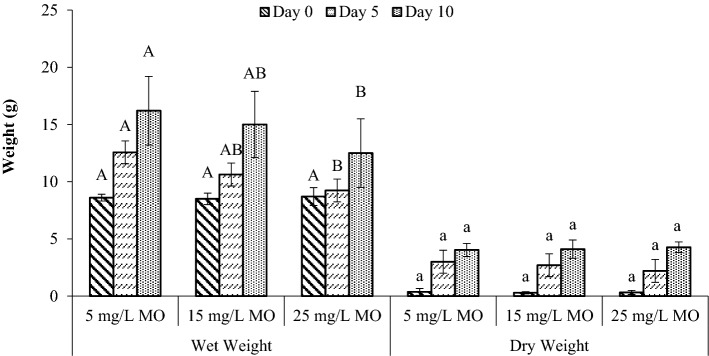
Effect of MO dye on *S. molesta* growth. The letters “A” and “B” represent statistically significant differences in wet weight on a specific day when compared with different MO concentrations (*p* < 0.05). The letter “a” represents no statistically significant differences in dry weight on a specific day when compared with different MO concentrations. Values represent the mean of triplicate experiments.

**Figure 3:**
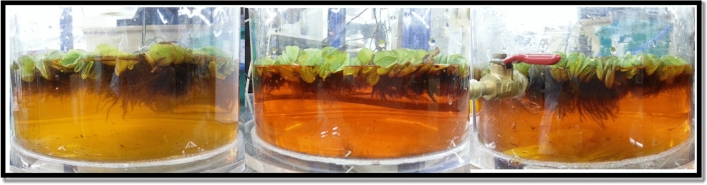
Physical appearance of *S. molesta* after a 10-day exposure to (a) 5 mg/L MO, (b) 15 mg/L MO, and (c) 25 mg/L MO.

Plant growth was observed for 10 days of MO exposure. As illustrated in Fig. 2, the wet and dry weight of *S. molesta* can grow well in all concentrations, while better growth was physically observed (Fig. 3) for lower MO concentrations (5 mg/L). As shown by the results, all of the plants had increases in wet weight after exposure to the MO dye contaminants. The wet weight increased from 8 ± 0.5 g to 14 ± 2 g in different MO dye concentrations of aqueous solution. At the end of 10-day exposure, the leaves of *S. molesta* remained green which contributed to the removal performance during the operation period, confirming the capability of *S. molesta* to treat dye-contaminated water. According to the two-way ANOVA, there was a significant difference (*p* < 0.05) in wet weight with different MO concentrations, as depicted in Fig. 2. However, no statistically significant difference was found for dry weight at different MO concentrations (*p* > 0.05) (Fig. 2). The *F*-value for Levene’s test is 0.011 with *p* = 0.989, leading to retain the null hypothesis (no difference) for the assumption of homogeneity of variance. According to Dhir, Sharmila and Saradhi^38^, the roots of *Salvinia* species were able to store high amount of heavy metals than its leaves.

**Figure 4:**
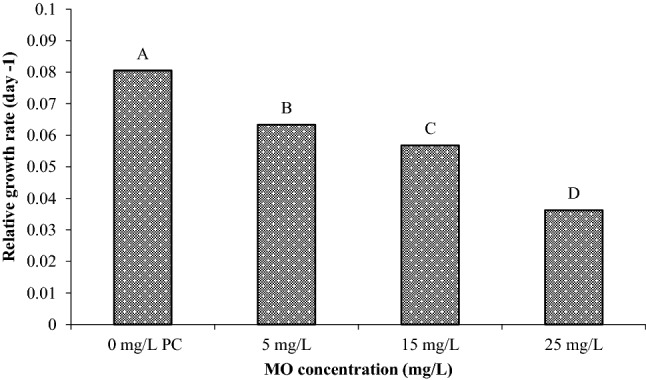
Effect of three MO dye concentrations (5, 15, and 25 mg/L) on the relative growth rate of *S. molesta.* There is no significant difference between relative growth rate (RGR) with the same letter (A or B).

The relative growth rate, which is dependent on wet weight, was affected by three concentration values of MO dye (5, 15, and 25 mg/L) compared to the corresponding plant control. As shown in Fig. 4, when the concentration of MO dye increased, there was a clear decrease in RGR after a 10-day period due to the effect of the plant when exposed to the dye. Statistical analysis results showed that the RGR had significant difference (*p* < 0.05) within the MO concentrations, confirming that all MO concentrations can grow and be regenerated although RGR decreases. Based on the dye concentration as tabulated in Table 2, the ratio of dye content to the fresh biomass of plants can be calculated as mg dye per fresh wet weight of plants to determine the amount of biomass required to remove certain amount of dye in water. According to Al-Badawi et al.^39^ findings, the ratio of plant numbers to the total mass of contaminant should be calculated to determine the phytotoxicity effects of the contaminant concentration. The same principle can be used if this plant were to be applied in real wastewater of different concentrations. In this work, the ratio was determined as the amount of initial dye content to the fresh wet weights since floating plants were used. From Table 2, a ratio of 0.67 mg MO mass / g of fresh plants would be the maximum ratio to be used to quantify the amount of required plant biomass for future application in different dye concentration of wastewater or in pilot scale application.

**Table 2:** Ratio of initial dye content in contaminated water to the fresh wet weight of *Salvinia molesta.*

Dye concentration (mg/L MO)	5	15	25
Volume of wastewater (L)	4	4	4
Initial dye content (mg)	20	60	100
Fresh wet weight (g)	150	150	150
Ratio of dye to wet weight (mg/g)	0.13	0.40	0.67

Hence, the overall findings indicate that *S. molesta* has potential used in phytoremediation and can be applied in wastewater treatment ponds with low pollutant concentrations, which is in agreement with the conclusion obtained by Muthunarayanan et al.^40^, who used *Eichhornia crassipes* to decolourise dyes in textile effluent. In addition, *S. molesta* had demonstrated to remove 31% of ammonia after 12 day of exposure^41^.

### Decolorization of methyl orange dye

**Figure 5:**
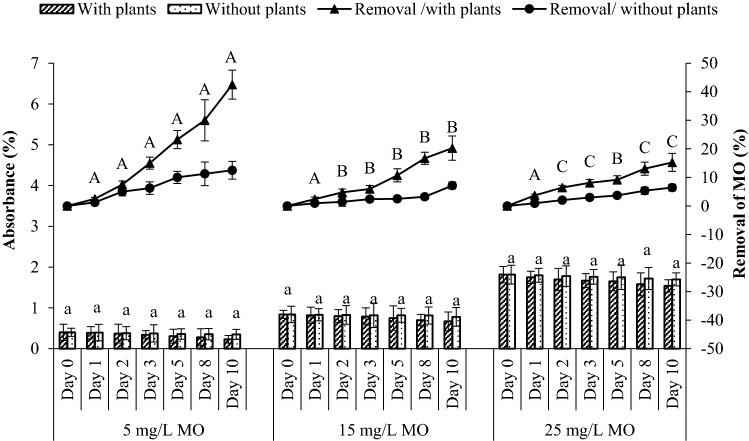
Effect of *S. molesta* on the decolorization of MO dye at different concentrations (5, 15, and 25 mg/L). Letter “a” means significant decrease in MO concentration between the two systems. Letters A, B, and C represent statistically significant differences in removal efficiency of MO from water within MO concentrations for each day (*p* < 0.05). Mean ± SD (n = 3).

Removal of dye was determined at different concentrations with 150 g of *S. molesta.* The adsorption of the three MO dye concentrations within 10 days varied greatly compared to the control dye (without plants) (Fig. 5). After a 10-day exposure of *S. molesta* to 5, 15, and 25 mg/L MO dye, the concentrations of the dye decreased to 2.9, 8.95, and 20.92 mg/L compared to the glass vessel without plants, in which the MO dye decreased to 4.6, 10.5, and 23.12 mg/L, respectively. The MO decolorization efficiencies for different concentrations (5, 15 and 25 mg/L dyes) were 42, 20, and 15%, respectively, indicating the dye can be removed effectively by *S. molesta*. The decolorization of the MO dye in the contaminant control was only 12, 7, and 6% for 5, 15, and 25 mg/L dye concentrations, respectively, providing evidence for the significant role of the plants in the removal of MO dye. Extending treatment time may lead to better dye removal achievement. Study on different type of aquatic plants indicated same trend as obtained by Yaseen and Scholz^37^, who utilised floating plant of *Lemna minor* in microcosms as a polishing stage for treatment of 10 mg/L dye in textile wastewater with 53% removal. Statistical analysis confirmed that there was a significant difference (*p* < 0.05) in MO removal within the different concentrations for each day, as shown in Fig. 5. The decolourization in the control glass vessel might be due to the microbe activities and photodegradation^25^.

Comparing the results of the current study with previous studies, a study by Li et al.^42^ showed a new novelty for the decolorization of the azo methyl orange (MO) dye in aqueous solution through a new clay-supported nanoscale zero-valent iron with 99.1% decolorization over 50 min. Ornamental plants of *Tagetes patula*, *Aster amellus*, *Portulaca grandiflora* and *Gaillardia grandiflora*, grown for one month on the ridges of wetland which was irrigated with textile wastewater, could reduce the ADMI values in soil by 18, 25, 40, 47, 66 and 73% as observed within 30 days, respectively^43^. Plants of *Fimbristylis dichotoma* and *Ammannia baccifera* had decolorized 50 mg/L methyl orange up to 91% and 89% after 60 h exposure, respectively. Whereas, when applied together as a consortium, MO decolorization of 95% was achieved within 48 h of exposure^44^. Also, Chandanshive et al.^45^ observed that *Salvinia molesta* had the capability to degrade azo dye, Rubine GFL, up to 97% at a concentration of 100 mg/L within 3 days using 6,072 g of root biomass.

As shown in Table 3, there was a significant interaction between treatment, time and MO concentrations with removal of MO efficiency by *S. molesta* [F(72) = 59.603, *p* < 0.05]. While, there was no significant interaction between time and MO concentrations with plant growth, F(18) = 2.359, *p* > 0.05.

**Table 3:** Interaction between dependent variables of removal efficiency and plant growth (wet weight) with independent variables of treatment, time, and MO concentrations.

Dependent variable	Tests of between-subjects effects			
	Source	df	F	Sig.*
Removal efficiency	Treatment * time	5	316.071	0.000
	Treatment * MO	2	246.161	0.000
	Time * MO	10	140.802	0.000
	Treatment * time * MO	10	59.603	0.000
Plant growth (wet weight)	Time * MO	4	2.359365	0.092

*Difference is significant at the 0.05 level.

### Fourier transform infrared (FTIR) spectroscopy analysis for phytotransformation

**Figure 6:**
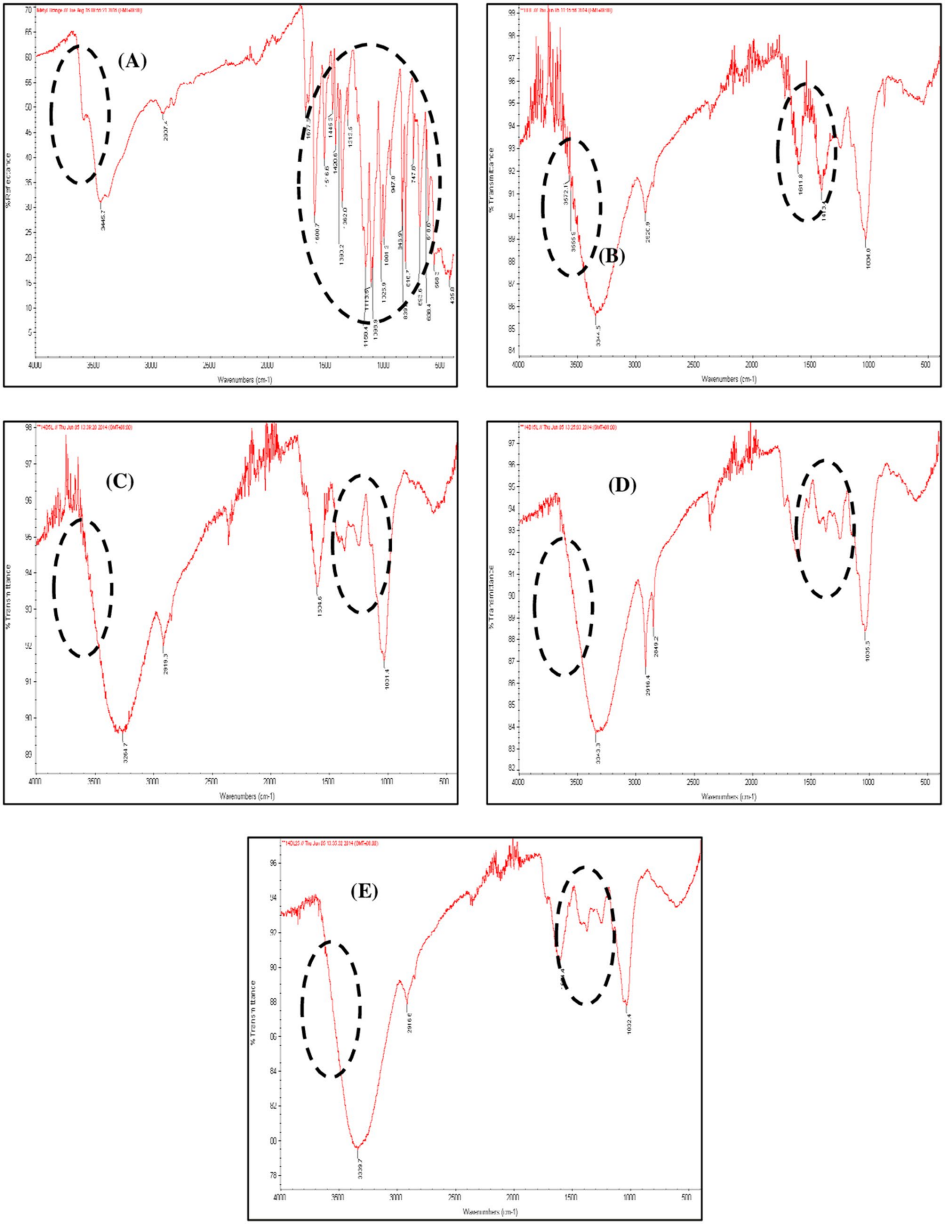
Fourier transform infrared (FTIR) spectral analysis of the samples: (A) MO dye, (B) *S. molesta* planted in distilled water, (C) *S. molesta* in 5 mg/L MO, (D) *S. molesta* in 15 mg/L MO by *S. molesta*, and (E) *S. molesta* in 25 mg/L MO. The dashed black circles focus on the change in compounds in different water media.

The FTIR spectrum of the control dye (Fig. 6A) varied significantly from the spectrum of MO decolourization at different concentrations by *S. molesta* (Fig. 6C–E), and it was found that *S. molesta* that grow in distilled water (Fig. 6B). The results support the phytotransformation of the dye into different metabolites. The FTIR spectrum of the MO dye (Fig. 6A) shows the presence of different high peaks at 3,445.7 cm^−1^ for the secondary amides (N–H stretch), 1,600.6 cm^−1^ for the aromatic compounds (C–C stretch), 1,113.5 cm^−1^ for aliphatic amines (C–N stretch), and 693.6 cm^−1^ for alkyl halides (C–Cl stretch).

The FTIR spectra of MO phytotransformation at different concentrations by *S. molesta* (Fig. 6C–E) show major peaks at 3,338.8 cm^−1^ for secondary amides (N–H stretch); 2,917 cm^−1^ for alkanes (C–H stretch); 2,849.2 cm^−1^ for aldehydes (H–C = O: C–H stretch); 1616.5 cm^−1^ for amines (N–H bend); and 1,033.8 cm^−1^ for alcohols, carboxylic acids, esters, and ethers (C–O stretch). While the FTIR spectra of the *S. molesta* plant in distilled water (Fig. 6B) showed peaks at 3,572 cm^−1^ for alcohols (O–H), 2,920.9 cm^−1^ for alkanes (C–H stretch), 1611.8 1 cm^−1^ for amines (N–H bend), 1,413.4 cm^−1^ for aromatics (C–C stretch), and 1,034 cm^−1^ for aliphatic amines (C–N stretch), indicating substantial changes in the peak position in comparison to the exposed plants with MO dye (Fig. 6C–E). The disappearance of a peak that was present in the spectrum of the dye indicates that the MO dye bond split^46^.

## Conclusions

The outcomes have indicated the significant role of the floating plant, *S. molesta* for decolorization of 5 mg/L of MO dye, with a removal efficiency of 42% compared with only 12% for the control MO dye over 10 days. In addition, FTIR tests confirmed the phytotransformation of MO dye by *S. molesta.* Since green biomass can be regenerated as wet weight increased from 8 ± 0.5 g to 14 ± 2 g in different MO dye concentrations of aqueous solution and is not affected by MO dye, phytoremediation using *S. molesta* is a promising approach to remove dyes from contaminated water.
